# Harnessing Large Cohorts and AI to Bridge Genomic Discovery and Clinical Practice

**DOI:** 10.1093/gpbjnl/qzaf104

**Published:** 2025-11-27

**Authors:** Bitao Zhong, Shaoqi Wang, Xiaoxi Jing, Aniruddh P Patel, Yajie Zhao, Minxian Wang

**Affiliations:** China National Center for Bioinformation, Beijing 100101, China; Beijing Institute of Genomics, Chinese Academy of Sciences, Beijing 100101, China; China National Center for Bioinformation, Beijing 100101, China; Beijing Institute of Genomics, Chinese Academy of Sciences, Beijing 100101, China; University of Chinese Academy of Sciences, Beijing 100049, China; Changping Laboratory, Beijing 102206, China; Heart and Vascular Institute, Massachusetts General Hospital, Boston, MA 02114, USA; Cardiovascular Research Center and Center for Genomic Medicine, Massachusetts General Hospital, Boston, MA 02114, USA; Program in Medical and Population Genetics, Broad Institute of Harvard and MIT, Cambridge, MA 02114, USA; Changping Laboratory, Beijing 102206, China; China National Center for Bioinformation, Beijing 100101, China; Beijing Institute of Genomics, Chinese Academy of Sciences, Beijing 100101, China; University of Chinese Academy of Sciences, Beijing 100049, China

Large-scale population cohorts have become a cornerstone of modern biomedical research, enabling systematic discovery of disease mechanisms, refining risk stratification, and advancing precision medicine into reality. By integrating multi-omics and deep phenotypic data, these resources provide a powerful foundation for uncovering biological drivers of health and disease, guiding prevention strategies, and improving population health outcomes. In the current era marked by the rapid emergence of artificial intelligence (AI), data from large-scale population cohorts are becoming increasingly indispensable. Despite remarkable progress, population-scale studies still face fundamental challenges, including limited diversity, inequitable representation, incomplete coverage of certain omics layers, and persistent barriers to effective data sharing across cohorts, and challenges in applying the most advanced analytical tools and AI-driven methods to cohort studies. This special issue, “New Era of Large-cohort Studies”, seeks to address these gaps and showcase recent advances. It includes 15 studies that collectively highlight advances spanning data governance and cohort infrastructure, large-scale genetic discovery across ancestries, especially the East Asian ancestry, and the use of population diversity to advance biomedical research. These studies provide insights into complex diseases and introduce computational innovations that enhance risk prediction and molecular characterization. Meanwhile, new population resources further strengthen the foundation for inclusive and equitable precision medicine. Overall, this special issue illustrates the vital role of diverse, well-curated cohort resources in accelerating genomic discovery and risk stratification, and in fostering global collaboration toward more equitable and actionable solutions for precision medicine.

## The challenges and solutions for cohort data sharing

Our special issue kicked off with a perspective on data sharing. Leveraging cohort-building experience, Jie Song et al. [1] addressed the challenges and solutions for sharing genomic data from large cohort studies, and highlighted prohibitive financial costs, technical infrastructure demands, and complex ethical and legal regulations as significant barriers. Instead of a simple data release, they propose a collaborative ecosystem for data sharing. Key solutions include tiered cost-sharing models for sustainability, federated data systems for security and cross-border compliance, and transparent governance to ensure fair access and resolve conflicts over intellectual property. The central argument is that unlocking the value of cohort data requires shared responsibility among all stakeholders to build an open, equitable, and sustainable environment for sharing.

## New tools and resources for supporting East Asian-centric large cohort research

Methodological innovation and new datasets are essential for modern genomic cohort studies, as these investigations generate data at scales and complexities that demand sophisticated computational tools and platforms. Without such infrastructure, it would be impossible to integrate, analyze, and extract meaningful biological insights from the vast, multidimensional datasets generated by large cohorts. The studies presented in this issue highlight new tools explicitly developed to address these analytical challenges.

Shujia Huang et al. [2] introduce GDBIG, the first Chinese birth cohort genomic platform, built on low-depth whole-genome sequencing (WGS) data from 4053 participants in family units. It addresses the lack of genomic resources for Asian birth cohorts and provides a user-friendly platform that integrates several key tools: a variant browser for allele frequencies, a family-sample-enriched genotype imputation server, and interfaces for genome-wide association study (GWAS) meta-analysis of pregnancy-related traits.

Regarding the imputation of rare variants, Jinglan Dai et al. [3] used WGS data from 150,119 UK Biobank individuals as a ground truth to demonstrate that a large reference panel can effectively impute rare variants, thereby improving the statistical power of rare variant association studies.

While Weijie Zhang et al. [4] constructed a comprehensive database dedicated to highland human populations, beyond the imputation service, also including visualization of phenotypes by altitude and sex, exploration of genomic diversity and natural selection signatures, and curated GWAS results. This resource serves as a vital platform for research on human adaptation to hypoxic environments.

Linhai Xie et al. [5] developed the Survival Reinforced Patient Stratification (SRPS) algorithm, a transfer-learning approach that adapts known molecular subtypes from a discovery cohort to new, unlabeled cohorts while preserving prognostic and molecular characteristics. SRPS uses a dual learning strategy: supervised learning on subtype labels in the source cohort and reinforcement learning using survival data in the target cohort to maximize prognostic discrimination. Using this newly developed algorithm, peptidylprolyl isomerase C was identified as a key biomarker for poor-prognosis hepatocellular carcinoma.

Zheng Dong et al. [6] employed whole-genome bisulfite sequencing (WGBS) to identify co-methylated regions (CMRs) in lymphoblastoid cell lines from individuals of European and African ancestry. It identified 101 population-specific CMRs, of which 91 were novel compared with previous array-based studies. Over half of these population-specific CMRs were associated with nearby genetic variants, and their methylation differences were larger when linked to single-nucleotide polymorphisms with significant differences in allele frequency between populations. The population-specific methylation patterns were also validated in an East Asian cohort and in peripheral blood samples, demonstrating their broader relevance across populations and tissues.

Yuanxin Wang et al. [7] conducted a systematic analysis of 7331 samples, which revealed 7216 high-quality mitochondrial DNA (mtDNA) variants classified into 22 macrohaplogroups and 1466 nuclear mtDNA segments (NUMTs). Additionally, they identified 88 mtDNA variants and 642 NUMTs that were found only in the NyuWa Chinese population. This research provides a comprehensive resource of Chinese mtDNA variations, highlighting population-specific genetic features and their potential implications for mtDNA-related diseases.

Collectively, these platforms and tools significantly enhance data accessibility, analytical power, and reproducibility for the East Asia-centric research community.

## Biological insights from large cohort studies

A key focus of this issue is the use of genetic and integrated multi-omics approaches to unravel the biological mechanisms underlying complex diseases. Studies employ GWAS, WGS, and functional follow-up to identify novel susceptibility loci and prioritize causal genes for downstream translational studies.

Mengying Wang et al. [8] performed WGS on 10,763 Chinese individuals from the Healthy Zhejiang One Million People Cohort (HOPE Cohort) to identify genetic factors associated with subjective cognitive decline (SCD). The analysis revealed novel associations with SCD involving rare variants in *SEPHS2* and *CLVS2*, and eight known loci related to cognitive decline were also validated.

Yu Qian et al. [9] identified 92 independent loci through a GWAS of 11 dual energy X-ray absorptiometry (DXA)-derived bone mineral density traits in over 30,000 individuals of European ancestry. Polygenic risk score (PRS) was associated with fracture risk independent of clinical factors. Furthermore, the authors prioritized druggable targets such as *ESR1* and *SREBF1*, highlighting potential opportunities for drug repurposing.

Yifan Du et al. [10] conducted a large-scale GWAS meta-analysis of aortic aneurysm and dissection (AAD) by combining its subtypes, analyzing 11,148 cases and 708,468 controls of European ancestry. It identified 24 susceptibility loci, including four novel loci, and prioritized 53 candidate genes through integrative multi-omics approaches. Cell-type-specific analyses highlighted arterial tissues as key sites of action. Functional experiments validated the roles of prioritized genes in vascular smooth muscle and endothelial cell functions, providing insights into AAD mechanisms and therapeutic strategies.

Xingzhong Zhao et al. [11] improved the estimation of brain age from magnetic resonance imaging (MRI) scans by employing an adversarial convolutional network on UK Biobank data. Their analysis revealed that the heritability of brain age gap (BAG) is enriched in regulatory regions and glial cells. Furthermore, they prioritized BAG-associated genes implicated in epigenetic aging and identified hub transcription factors that regulate pleiotropic risk genes associated with diverse brain disorders.

These findings collectively provide key genetic insights into the biological mechanisms underlying a spectrum of complex diseases, including cognitive decline, bone mineral density, aortic aneurysm, and brain aging. Using GWAS, WGS, and multi-omics integrating approaches, these studies identified novel susceptibility loci and prioritized causal genes and druggable targets, thereby laying the foundation for translational research and precision medicine.

## Insights into risk stratification and public health from cohort studies

The translation of genomic discoveries into clinical practice is a key goal for cohort studies. Studies in this issue also demonstrate that integrated risk models, combining polygenic and other molecular markers, are bridging this gap, informing patient stratification, and enabling innovative preventive medicine.

Yaning Zhang et al. [12] developed a multi-omics risk model for overweight-related hypertension (OrH) in 7605 Chinese individuals. By systematically comparing PRSs and methylation risk scores (MRSs), they found that integrating both into a single multi-omics model significantly improved prediction accuracy for obesity, hypertension, and OrH. The results highlight the value of multi-omics approaches for personalized risk assessment.

Yuexin Zhu et al. [13] investigated the benefits of cardiovascular health for calcific aortic valve stenosis (CAVS) risk, stratified by polygenic risk and lifestyle, in 153,312 UK Biobank participants. It found that higher Life’s Essential 8 (LE8) scores (better lifestyles) were independently associated with lower CAVS risk, whereas higher genetic risk was associated with higher disease risk. A significant additive interaction between genetic risk and lifestyle was found. The study highlights that optimizing lifestyle behaviors can mitigate genetic risk for CAVS.

Fengzhe Xu et al. [14] studied the molecular mechanisms linking statin use to an increased risk of type 2 diabetes (T2D) by analyzing multi-omics data (gut microbiome, blood metabolites, and proteins) from East Asian and European populations. Using genetic variants to mimic statin effects, specific biomarkers altered by statin therapy were identified. A key finding was that statins increase gastric inhibitory polypeptide (GIP) levels, a protein that, in turn, increases T2D risk.

Jinyeon Jo et al. [15] analyzed the genetic basis of obesity and its comorbidities in 93,673 Koreans using GWAS and PRSs. Their analyses identified genome-wide significant loci and demonstrated that East Asian populations showed stronger genetic correlations between abdominal obesity and obesity-related diseases. Both single-trait and multiple-trait PRSs stratified individuals by disease risk, with the multiple-trait model performing better. The study highlights the value of multiple-trait PRS models and underscores the importance of ancestry-specific genetic research in addressing the obesity epidemic.

These studies showcase the translational power of multi-omics and genetic risk prediction, demonstrating how integrating polygenic and methylation risk scores enhances disease risk prediction, how optimal lifestyle behaviors can mitigate high genetic risk for complex diseases, and how genetic analyses can uncover the molecular pathways underlying medication side effects, collectively charting a course toward more precise and actionable clinical interventions.

In summary, the studies in this special issue collectively exemplify the complete cycle of the large cohort studies, spanning from foundational resource development and data governance to mechanistic insights and clinical translation. These contributions not only provide critical datasets, tools, and key resources but also deepen our understanding of disease genetics, thereby advancing the development of precise prevention strategies and innovative therapeutic approaches. The integration and progress showcased here mark a shift toward a more systematic and collaborative discipline, laying a robust scientific foundation for improving human health by fully leveraging large cohort studies.

## CRediT author statement

**Bitao Zhong:** Writing – original draft, Writing – review & editing. **Shaoqi Wang:** Writing – original draft, Writing – review & editing. **Xiaoxi Jing:** Writing – review & editing. **Aniruddh P. Patel:** Conceptualization, Writing – review & editing. **Yajie Zhao:** Conceptualization, Writing – review & editing. **Minxian Wang:** Conceptualization, Writing – review & editing. All authors have read and approved the final manuscript.
